# Next generation of cancer immunotherapy calls for combination

**DOI:** 10.18632/oncoscience.343

**Published:** 2017-03-31

**Authors:** Paola Cappello, Francesco Novelli

**Affiliations:** Department of Molecular Biotechnology and Health Sciences, University of Turin; Center for Experimental Research and Medical Studies, AOU Città della Salute e della Scienza di Torino; Molecular Biotechnology Center, University of Turin, Turin, 10126, Italy; Department of Molecular Biotechnology and Health Sciences, University of Turin; Center for Experimental Research and Medical Studies, AOU Città della Salute e della Scienza di Torino; Molecular Biotechnology Center, University of Turin, Turin, 10126, Italy

**Keywords:** pancreatic ductal adenocarcinoma, immunotherapy, tumor associated antigens (TAA), α-enolase (ENO1), fibrosis

Pancreatic ductal adenocarcinoma (PDA) is a lethal cancer resistant to chemotherapy and radiation therapy, thus requiring novel therapeutic options. Although immunotherapy has been widely explored for cancer treatment, pancreatic cancer seems to be not suitable for this approach as it is considered an “immune privileged site”. This is due to an immunosuppressive environment or to a low rate of mutations that generate neo-antigens able to activate anti-tumor T cells. However, our data demonstrate the presence of antigen-specific T cells in PDA, which recognize the tumor associated antigen (TAA) alpha-enolase (ENO1) [1], and could be challenged and expanded by a vaccine approach. This hypothesis was verified using a DNA vaccine coding for ENO1 in a genetically engineered mouse model (GEM) that spontaneously develops PDA. ENO1-vaccinated mice showed prolonged survival associated with the induction of specific antibodies to ENO1 that activate complementdependent cytotoxity (CDC); the decrease in tumor infiltration by T regulatory cells and myeloid-derived suppressor cells (MDSC); as well as the concomitant increase in tumor infiltrating T cells [2] re-localized into tertiary lymphoid tissue, reminiscent of a germinative center [3]. In addition to the induction of CDC, anti- ENO1 antibodies are also responsible for some other effects; firstly, the inhibition of migration and invasion of PDA cells, as demonstrated *in vitro* and *in vivo* [4], and secondly, the decreased infiltration of MDSC. Indeed, we observed that MDSC express high surface levels of ENO1 when stimulated by inflammatory stimuli, and anti-ENO1 antibodies were able to block the interaction of MDSC with endothelial cells, limiting their invasion into the tumor [5].

The goal of immunotherapy is to enhance and direct the body's immune system into recognizing tumor-specific antigens and thus mount an attack against the disease. In this light, the induction of a curative, long-lasting ENO1- specific humoral and cellular anti-tumor responses, such as that directed against other antigens, relies on the combination with adjuvants acting as immune-stimulatory agents (e.g. CD40) or limiting immunosuppression (immune check-point inhibitors, myeloid suppressor cell depletion). The recognition of tumor cells by the immune system begins with the release of TAAs by dying tumor cells, subsequently taken up from resident macrophages or dendritic cells (collectively called antigen-presenting cells, APC), which process them via membrane-bound vesicles into smaller peptides. These peptides are subsequently associated with the major histocompatibility complex (MHC) class II molecules, and presented to CD4 T cells, along with MHC class I molecules, and cross-presented to CD8 T cells. CD4 T cell activation is mandatory for both B cell activation and production of antibodies, as well as for cytotoxic T lymphocyte generation and maintenance.

In both orthotopic and GEM mouse models of PDA, we recently demonstrated that genetic or pharmacological inhibition of PI3Kγ limits myeloid suppressor cell infiltration, skews macrophage activation to the antitumoral function and delays tumor growth and metastasis (particularly in combination with chemotherapy) by increasing the number of T cells that infiltrate the tumor [6]. This observation gives rise to the promising idea of targeting myeloid suppressor cells to limit tumor progression, even if the eventual recurrence could be specifically counteracted with the induction of the adaptive anti-tumor immune response achievable by vaccines. Other potential approaches of targeting myeloid cells in PDA have been shown by targeting CD40 or colony stimulating factor (CSF)1/CSF1 receptor (R). Anti-CD40 agonists redirect tumor-infiltrating monocytes/macrophages to induce degradation of fibrosis via metalloproteases, without T cell involvement [7]. CSF1/CSF1R blockade has been demonstrated to skew infiltrating macrophages from a more suppressive alternative phenotype, to an antitumoral phenotype, with the consequent activation of T cells, thus proving beneficial in the combination with PD1 or CTLA4 treatment [8].

All these approaches are promising for their combination with active immunotherapy. They have already shown clinical potential in combination with chemotherapy, but with vaccines have to be explored yet. There is an urgent need in cancer to avoid recurrence and metastasis in general and, therefore, active immunization against TAAs represents the only effective chance with low side effects. However, pre-clinical and clinical studies must elucidate the right timing, doses and administration modes for these candidate combinations to obtain the best benefits.
